# Effect of Depth of Flooding on the Rice Water Weevil, *Lissorhoptrus oryzophilus*, and Yield of Rice

**DOI:** 10.1673/031.013.6201

**Published:** 2013-06-25

**Authors:** Kelly V. Tindall, John L. Bernhardt, Michael J. Stout, Donn H. Beighley

**Affiliations:** 1 University of Missouri, Division of Plant Sciences, P.O. Box 160, Portageville, MO 63873, USA; 2 University of Arkansas, Department of Entomology, Rice Research and Extension Center, 2900 Hwy. 130 East, Stuttgart, AR 72160, USA; 3 Department of Entomology, Louisiana Agricultural Experiment Station, Louisiana State University Agricultural Center, 402 Life Sciences Building, Baton Rouge, LA 70803, USA; 4 Southeast Missouri State University, Department of Agriculture, Southeast- Maiden, 700 N. Douglas St., Maiden, Missouri 63863, USA; 5 DuPont Pioneer, 2223 Old Troy Rd., Union City, TN 38261

**Keywords:** cultural control, *Oryza sativa*, rice water weevil management

## Abstract

The rice water weevil, *Lissorhoptrus oryzophilus* (Kuschel) (Coleoptera: Curculionidae), is a semi-aquatic pest of rice and is the most destructive insect pest of rice in the United States. Adults oviposit after floods are established, and greenhouse studies have shown that plants exposed to deep floods have more eggs oviposited in leaf sheaths than plants exposed to a shallow flood. Experiments were conducted in three mid-southern states in the USA to determine if the depth of flooding would impact numbers of *L. oryzophilus* on rice plants under field conditions. Rice was flooded at depths of approximately 5 or 10 cm in Arkansas in 2007 and 2008 and Louisiana in 2008, and at depths between 0–20 cm in Missouri in 2008. Plants were sampled three and four weeks after floods were established in all locations, and also two weeks after flood in Missouri. On all sampling dates in four experiments over two years and at three field sites, fewer *L. oryzophilus* larvae were collected from rice in shallow-flooded plots than from deep-flooded plots. The number of *L. oryzophilus* was reduced by as much as 27% in shallow-flooded plots. However, the reduction in insect numbers did not translate to a significant increase in rice yield. We discuss how shallow floods could be used as a component of an integrated pest management program for *L. oryzophilus.*

## Introduction

Rice crop production is a unique system, because in most rice-producing regions of the world, rice is grown as a lowland crop in which fields are flooded for the majority or the entirety of the growing season. The primary motive for flooding is weed control; however, water management in rice fields also impacts insect pests. For example, in the southern USA, flooding rice fields helps control terrestrial pests like fall armyworm, *Spodoptera frugiperda* J.E. Smith, and chinch bug, *Blissus leucopterus leucopterus* Say (Hummel et al. 2009). Conversely, many of the most important pests of rice are aquatic or semi-aquatic, and flooding fields can intensify problems with these pests.

One example of a semi-aquatic pest for which flooding intensifies problems is the rice water weevil, *Lissorhoptrus oryzophilus* (Kuschel) (Coleoptera: Curculionidae) (Rice et al. 1999; Zou et al. 2004). *L. oryzophilus* is the most destructive insect pest of rice in the USA (Way 1990). This insect is native to the USA, but has invaded important rice-growing areas in Asia and Europe over the past 60 years, and thus now poses a global threat to rice production (Saito et al. 2005). Adults feed on rice leaves and are often found in non-flooded fields, but adult feeding is generally not economically damaging except under unusually heavy infestations. Flooding acts as a trigger for oviposition, and once a flood is established, females oviposit in leaf sheaths beneath the water surface (Stout et al. 2002b). Eggs eclose and larvae move to the roots and begin feeding in or on roots. The insects pass through four instars and a pupal stage in approximately 30 days on roots (Zou et al. 2004). Small-plot research and sampling of commercial fields indicated yield losses from root feeding by *L. oryzophilus* larvae would likely exceed 10% in many areas if no control measures were taken.

Insecticides have been the primary means of control of *L. oryzophilus* for many years. The larvicide carbofuran was a viable chemical control option for more than 30 years until the mid 1990s, when the Environmental Protection Agency began a gradual phase-out of carbofuran use in rice (Stout et al. 2000). Following this regulatory action, carbofuran was replaced by synthetic pyrethroids (lambda-cyhalothrin and others), an insect growth regulator (diflubenzuron), and a larvicidal seed treatment (fipronil). The manufacturer of fipronil voluntarily removed this insecticide from the USA rice market in the early 2000s (Bennett 2004). Until the seed treatments chlorantraniliprole (DuPont 2010) and thiomethoxam (EPA 2009) were approved from 2008–2010, application of synthetic pyrethroids was the dominant tactic used for *L. oryzophilus* management in the U.S.

In addition to chemical control options, several cultural control measures for *L. oryzophilus* have been investigated. Rice planted at low seeding rates can be more vulnerable to high weevil infestations (Thompson and Quisenberry 1995; Stout et al. 2009) and suffer greater yield losses than rice planted at higher seeding rates (Stout et al. 2009). Early planting dates in Louisiana appear to allow plants to escape severe weevil infestations sometimes and to tolerate damaging populations of *L. oryzophilus* better than at later planting dates (Thompson et al. 1994; Stout et al. 2011). However, Espino et al. (2009) suggested that producers in Texas must choose between planting dates that will produce higher yields or planting dates that produce low *L. oryzophilus* populations. There is evidence of differences in the tolerance of some rice varieties and lines to *L. oryzophilus* feeding (N'Guessan et al. 1994; Stout et al. 2001; Bernhardt and Richards 2003).

**Table 1.**
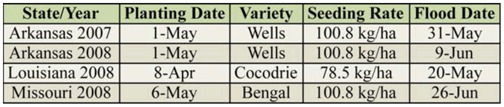
Agronomic information for depth of flooding experiments conducted at three sites over 2007 and 2008.

Several other cultural control measures involve managing water in ways that interfere with *L. oryzophilus* biology. Draining of fields infested with *L. oryzophilus* larvae to discourage further oviposition and possibly kill larvae established on roots has long been used. However, draining is not recommended, because rainfall can prevent the soil from drying sufficiently, and additional input costs are associated with draining (Hesler et al. 1992; Quisenberry et al. 1992; Thompson et al. 1994). Manipulating water such that plants are older when floods are established can aid in reduction of *L. oryzophilus* damage because older plants are better able to tolerate injury from weevil larvae than younger plants (Stout et al. 2002a) and are less preferred for oviposition (Bang and Tugwell 1976). Delayed flood can be accomplished by adopting drill-seeding practices instead of water-seeding rice, or delaying flooding in water-seeded rice from the 2–3 leaf stage to the 4–5 leaf stage (Rice et al. 1999; Zou et al. 2004; Bernhardt 2006). The recent introduction of herbicide-tolerant varieties has allowed more flexible water management (i.e., drill-seeded rice) (Rice et al. 1999; Tindall et al. 2004).

Another potential method for suppressing populations of *L. oryzophilus* that holds promise involves manipulating the depth of floods. Stout et al. (2002b) demonstrated the effects of flood depth on ovipositional preference of *L. oryzophilus* under semi-controlled conditions in a greenhouse. In no-choice experiments, the proportion of females that oviposited and the number of eggs oviposited increased as soil moisture/flood depth increased from dry soil to 5.1 cm of flood. Furthermore, free choice experiments showed that females had a stronger ovipositional preference for plants in a deep flood than for plants in saturated soil or a shallow flood. Stout et al. (2002b) suggested the manipulation of flood depth as a possible means of suppressing rice water weevils; however, this concept has not been tested under field conditions. In this paper, several field experiments that investigated the impact of a shallow flood verses a deep flood on *L. oryzophilus* infestations in the field are described.

## Materials and Methods

Studies were conducted at three sites in three mid-southern states in the USA to investigate shallow flooding as a potential means of reducing rice water weevil infestations. The Arkansas site was located at the Rice Research and Extension Center near Stuttgart (Arkansas County) on a Dewitt silt loam soil (fine, smectitic, Typic Albaqualtic). The study sites in Missouri and Louisiana were on Crowley silt loam at the Missouri Rice Research Farm near Glennonville (Dunklin County) and the LSU AgCenter Rice Research Station near Crowley (Acadia Parish). Experiments were conducted in 2007 and 2008 in Arkansas and in 2008 in Missouri and Louisiana. Table 1 provides agronomic information for each location. Plots were maintained according to state production guidelines, but no insecticides were used (Slaton 2000; Blanch et al. 2009; Stevens et al. 2011)

Plots were flooded 31 to 51 days after planting. When permanently flooded, plots were exposed to either a deep or shallow flood. The deep flood was approximately 10 cm, and the shallow flood was approximately 5 cm in Arkansas and Louisiana. In Missouri, the following range of flood depths were used: 0, 5, 10, 15, and 20 cm. Once a flood was established, flood depths were maintained by periodically adding or removing water to compensate for rainfall or evaporative loss. Flood depths were maintained all season in Missouri and Arkansas; in Louisiana, flood depths were maintained for 4 1/2 weeks, and then all plots were flooded to a depth of 10 cm until the field was drained for harvest. In Louisiana, plots measured 7.6 m × 27.4 m and were bordered on all sides by earthen levees. Each plot in Louisiana contained three subplots of rice measuring 1.2 m × 5.5 m. Plots had separate access to a lateral for irrigation, and plots were arranged in a randomized complete block with two treatments (shallow and deep flood) and three replications. In Arkansas, there were four replications of each treatment arranged as a randomized complete block design. Each plot measured 1.6 × 7.62 m and was surrounded by levees. The plots in Missouri were 1.3 m × 3.7 m, with three replications of each flood treatment arranged as a randomized complete block. Due to the intensity of water management and the spatial requirements of replicating the flood depth treatment, multiple tests were conducted within large bays (135 m × 5 m). One plot per bay was dedicated to this *L. oryzophilus* study, and there were three bays per flood depth.

Immature *L. oryzophilus* were sampled at two or more time points after flooding using a root-soil core sampler (9.2 cm diameter with a depth of 7.6 cm for cores pulled in Louisiana and Missouri, and 10.2 cm × 10.2 cm for cores pulled in Arkansas). Soil and larvae were washed from roots of plants into 40 mesh screen buckets or sieves. Buckets or sieves were immersed in a saturated saline solution, which caused larvae and pupae to float to the surface for counting (Smith and Robinson 1982). Three core samples per plot were taken in both Arkansas and Missouri, and 5 core samples were taken per subplot in Louisiana. Core samples were collected at 3 weeks after flood (WAF) and 4 WAF at all locations, and at 2 WAF in Missouri. In Missouri, core samples were not collected from the “0” flood depth at the 3 WAF sample date, because the ground was too hard to sample. Yields were taken using small-plot harvesters and converted to 12% moisture.

The number of *L. oryzophilus* immatures (larvae and pupae) at 3 and 4 WAF and yield data from Arkansas were analyzed separately for each sampling date using PROC MIXED for a randomized block design in SAS (SAS 2004), with “replication” and “replication (year)” in the RANDOM statement. Larval counts from the three subplots in each plot in the Louisiana experiment were averaged to obtain a mean value for each plot for each of the two sampling dates. These mean plot values were analyzed using PROC MIXED in SAS, with replication as a random effect and depth of flooding as a fixed effect. Core samples from each sampling date from Missouri were analyzed with PROC REG in SAS.

## Results

In Louisiana, the number of *L. oryzophilus* larvae and pupae at 3 WAF from plots with a shallow flood was approximately 20% lower than the number in the deep-flooded plots (Table 2). The number of weevils was 13% lower in the shallow-flooded plots than in the deep-flooded plots at the 4 WAF sampling point, but the difference was not significant. Grain yield was 19.1% higher in shallow-flooded plots compared to deep-flooded plots, although this difference was only marginally significant.

**Table 2.**
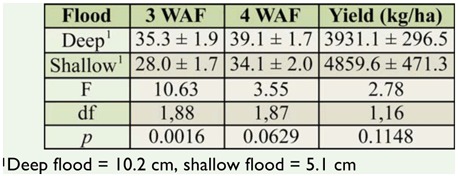
Mean number of immature *Lissorhoptrus oryzophilus* (larvae and pupae) per core sample 3 and 4 weeks after flood (WAF) from rice with deep and shallow floods, and corresponding yield, in Crowley, Louisiana, 2008.

The number of *L. oryzophilus* larvae and pupae from Arkansas in 2007 and 2008 followed a trend similar to that seen in Louisiana, and differences were not significant (Table 3). Samples from shallow-flooded plots had 27.6% and 25.7% fewer rice water weevils than samples from deep-flooded plots at 3 and 4 WAF, respectively, over the two years of the experiment. There was no significant difference in yield between the flood depths (Table 3).

Results from the Missouri experiment showed that the number of *L. oryzophilus* immatures recovered from core samples significantly increased as depth of flooding increased at 2 WAF (*F* = 27.25; df = 1,41; *p* < .0001; R^2^ = 0.3993). Although the trends with the number of *L. oryzophilus* at 3 and 4 WAF were similar to those at 2 WAF, differences among flood depths were not significant (3WAF: *F* = 2.37; df = 1,33; *p* = 0.1335; R^2^ = 0.0669; 4 WAF: *F* = 2.57; df = 1,42; *p* = 0.1163; R^2^ = 0.0577). Yield declined as depth of flooding increased, but the relationship was not significant (y = 10545 - 194.2x; *F* = 3.16; df = 1,13; *p* = 0.0987; R^2^ = 0.1957)

**Figure 1.**
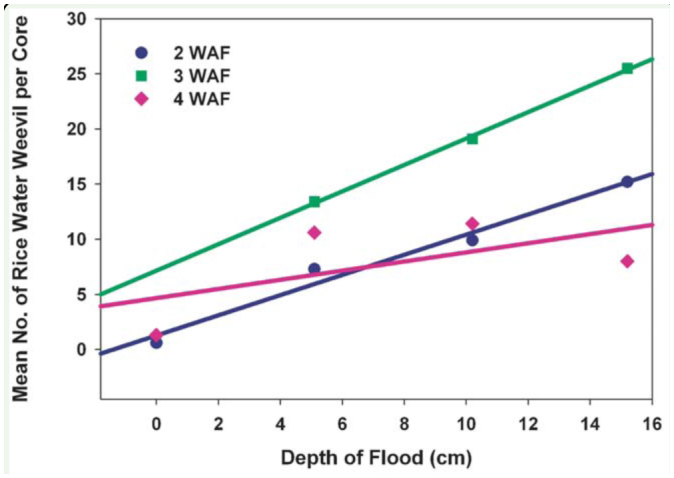
Densities of *Lissorhoptrus oryzophilus* in rice as a function of depth of flooding at 2, 3, and 4 weeks after flood (WAF), Glennonville, MO, 2008. (2 WAF: y = 0.301 + 2.836x - *F* = 27.25; df = 1,41; *p* < .0001; R^2^ = 0.3993; 3 WAF: y = 12.142 + 1.568x - *F* = 2.37; df = 1,33; *p* = 0.1 335; R^2^ = 0.0669; 4 WAF: y = 5.976 + 0.495X - *F* = 2.57; df = 1,42; *p* = 0.1163; R^2^ = 0.0577). High quality figures are available online.

**Table 3.**
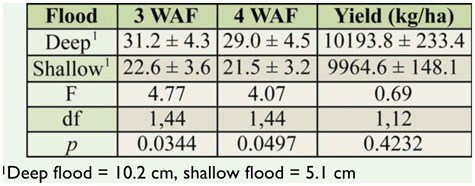
Mean number of immature *Lissorhoptrus oryzophilus* per core sample 3 and 4 weeks after flood (WAF) from rice with deep and shallow floods, and corresponding yield, in Stuttgart, Arkansas, USA, 2007 and 2008.

## Discussion

Prior greenhouse experiments showing an effect of flood depth on *L. oryzophilus* oviposition suggested that shallow flooding might be useful as a cultural control (Stout et al. 2002b). On all sampling dates in four experiments over two years and at three field sites, fewer rice water weevil larvae were collected from rice in shallow-flooded (5 cm) plots than from deep-flooded (10 cm) plots, although differences in larval number between the two treatments were not always significantly different. The number of weevils was reduced by as much as 27% in shallowflooded plots. In all cases, reductions in immature weevil densities were greater on earlier sampling dates (2 or 3 WAF) than later sampling dates (four WAF).

Considering the results of the prior greenhouse study by Stout et al. (2002), one possible mechanism for the reduction of larval number in shallow-flooded plots is reduced oviposition. In this previous study, under greenhouse conditions, more eggs were oviposited in plants flooded at a depth of 10.2 cm than in plants flooded at 5.1 cm in a choice test, but not in a no-choice test (Stout et al. 2002b), suggesting that the flood or a signal associated with the flood serves as stimulus for oviposition. However, reduced oviposition under shallow floods does not explain why reductions in larval populations were greater at earlier core sampling dates than at later core sampling dates in the present study, since differences in flood depths were maintained for at least 4 1/2 weeks in all experiments. One alternate explanation for the differences in immature weevil numbers between deep and shallow-flooded plots is that depth of flooding altered the abiotic environment of rice fields in a way that oviposition, egg hatching, and/or early-instar larval development were delayed in shallow-flooded plots relative to deep-flooded plots, thus accounting for the treatment differences at early but not later core samplings. For example, water temperature may have differed between plots flooded at different depths, and these temperature differences may have been responsible for delays in weevil development. However, water in shallow-flooded plots would be expected to be no cooler and probably warmer than water in deep-flooded plots, and under normal circumstances warmer water would be associated with more rapid insect development. A final possibility is that flooding induces responses in rice plants, and that the different flooding treatments induced these responses to different extents, thereby leading to different plant-mediated effects under the two treatments. With respect to this possibility, Stout et al. (2002) found that flooded plants were taller and had increased nutrients relative to unflooded plants. Further experiments will be needed to elucidate the mechanisms for reduced immature populations in shallowflooded plots.

A reduction in larval number did not translate to increases in yield in these experiments; however, there was a trend toward higher yields from shallow-flooded plots than from deep-flooded plots in studies from Louisiana and Missouri. Given the modest reduction in weevil numbers associated with shallow flooding and the non-significant increase in yield, it is clear that a shallow flood would not be a substitute for chemical control, but it may serve as a valuable component of an integrated pest management system. Other cultural practices that could be used in conjunction with a shallow flood include: avoiding planting at low seeding rates (Thompson and Quisenberry 1995; Stout et al. 2009), delaying the permanent flood to allow plants to develop a robust root system and become more tolerant to *L. oryzophilus* injury (Stout et al. 2002a), selecting a planting date appropriate to the area (Thompson et al. 1994; Espino et al. 2009; Stout et al. 2011), and choosing a variety that is more tolerant to weevil injury (N'Guessan et al. 1994; Rice et al. 1999; Stout et al. 2001; Bernhardt and Richards 2003; Tindall et al. 2004). A shallow flood is more likely to be beneficial in areas that have historically low infestation levels. For example, in the northern areas of rice production (Missouri, Arkansas, and Mississippi, USA), *L. oryzophilus* infestations are not as severe as in more southern areas, such as south Louisiana, USA (Stout et al. 2009).

Challenges may arise with maintaining shallow floods in commercial rice fields. More frequent watering may be necessary to prevent complete water loss from evaporation. Draining is not recommended for *L. oryzophilus* control, because there are costs associated with re-application of fertilizer due to volatilization of nitrogen when water is absent, additional herbicide needed to control weeds that germinate when fields are drained, and pumping of water for re-flooding (Hesler et al. 1992; Quisenberry et al. 1992; Thompson et al. 1994). There may be difficulties in maintaining a uniform flood of 5 cm on ground that has not been laser leveled. If an area of a field receives less water or the flood evaporates, the condition may lead to problems similar to those that occur when fields are drained. In addition to problems maintaining a shallow flood depth, the plant disease rice blast, *Pyricularia grisea*, tends to be less severe in flooded rice than dryland rice (Lai et al. 1999), and Cartwright and Lee (2000) suggested a consistently deep flood (≥ 10.2 cm) to avoid infestations of rice blast. Therefore, rice blast may be more problematic in shallow-flooded environments.

On the other hand, there are growing concerns of water resources for agricultural irrigation. The amount of water required by rice for normal plant functions is comparable to that required by other small grains. However, because of inefficiencies associated with water use in rice production, rice requires, on average, 2.5-fold more water than it actually uses (Bouman 2009). A shallow flood for *L. oryzophilus* suppression may have benefits beyond those associated with pest management if the amount of water used is reduced.

Given the potential positives and negatives associated with shallow flooding, an intermediate approach in which a shallow flood is implemented for a given period of time and a normal flood depth is applied thereafter may be useful. Similar to the benefits of a delayed flood (Stout et al. 2002a), a period of a shallow flooding may allow rice plants to escape heavy weevil pressure until plants are able to tolerate high numbers of weevils. A thorough examination of water use efficiency and an economic analysis will be necessary to determine costs associated with a shallow flood under large production systems.
